# MINE: a new way to design genetics experiments for discovery

**DOI:** 10.1093/bib/bbaf167

**Published:** 2025-04-16

**Authors:** Isaac Torres, Shufan Zhang, Amanda Bouffier, Michael Skaro, Yue Wu, Lauren Stupp, Jonathan Arnold, Y Anny Chung, H-Bernd Schuttler

**Affiliations:** Institute of Bioinformatics, University of Georgia, Athens, GA 30602 USA; Institute of Bioinformatics, University of Georgia, Athens, GA 30602 USA; Institute of Bioinformatics, University of Georgia, Athens, GA 30602 USA; Institute of Bioinformatics, University of Georgia, Athens, GA 30602 USA; Department of Genetics, Stanford University, Stanford, CA 94309 USA; Genetics Department, University of Georgia, Athens, GA 30602 USA; Institute of Bioinformatics, University of Georgia, Athens, GA 30602 USA; Plant Biology and Plant Pathology, University of Georgia, Athens, GA 30602 USA; Physics and Astronomy, University of Georgia, Athens, GA 30602 USA

**Keywords:** ensemble methods, MINE, mixed linear models, genetic networks, GWAS

## Abstract

The Maximally Informative Next Experiment or MINE is a new experimental design approach for experiments, such as those in omics, in which the number of effects or parameters *p* greatly exceeds the number of samples *n* (*p* > *n*). Classical experimental design presumes *n* > *p* for inference about parameters and its application to *p* > *n* can lead to over-fitting. To overcome *p* > *n*, MINE is an ensemble method, which makes predictions about future experiments from an existing ensemble of models consistent with available data in order to select the most informative next experiment. Its advantages are in exploration of the data for new relationships with *n* < *p* and being able to integrate smaller and more tractable experiments to replace adaptively one large classic experiment as discoveries are made. Thus, using MINE is model-guided and adaptive over time in a large omics study. Here, MINE is illustrated in two distinct multiyear experiments, one involving genetic networks in *Neurospora crassa* and a second one involving a genome-wide association study in *Sorghum bicolor* as a comparison to classic experimental design in an agricultural setting.

## Introduction

The classic approach to experimental design was developed by R. A. Fisher for linear models in 1935 and had a profound effect on all of science [1, 2]. Growing out of his work at the Rothamsted Experiment Station, he introduced widely the notion of precision of an experiment, randomization, ways of controlling heterogeneity through blocking and by the use of covariates, and the vast subject of experimental design in the context of linear models [3].

The focus of all of these efforts was not on discovery per se. Rather, the end goal was the precision of estimates and the power to test effects in a controlled experiment with the proper randomization and blocking practices in place. The number of replicates was such that the number of observations (*n*) was typically much greater than the number of effects (*p*) being estimated in the model. Unfortunately, this is no longer the typical situation of an omics experiment [4], such as a genome-wide association study (or GWAS) as an example. Instead, there may be only *n* = 1943 samples of sorghum accessions but over *p* = 400 000 potential effects of single nucleotide polymorphisms (or SNPs) on the complex trait of interest for an agronomic crop [5]. The methods of classic experimental design are limited to the situation of *n* > *p* for inference about model parameters and are not designed for data exploration for hypothesis generation.

While the original goal was the precision of estimates [2], the new goal is discovery. The reason that precision is less important is that the goal of such studies has shifted to the discovery of relationships in the data. The focus on precision of effects can only be addressed in follow-up studies when the relevant variables in the experiment have been identified and related. We desire to discover the appropriate nonlinear kinetic models that underly the biological clock at the molecular level [6] as we carry out a very expensive sequence of transcriptomic experiments. We wish to discover the relation of plant functional traits to SNPs in the nuclear genome or the assemblage of fungal symbionts in the microbiome most beneficial for plant growth [7–9] using models drawn from systems and population ecology [10]. How can the classic linear models [11] and newer models of systems biology [6] guide a discovery process, in which some GWAS studies have millions of SNPs, such as those on human height [12]?

A new approach to the design of large genomics experiments is introduced, one in which model-guided discovery is used adaptively [13] over time to find the variables that matter, considering a system in which the number of potential effects in the system (*p*) far exceeds the number of observations (*n*) on the system. The methodological approach utilizes ensemble methods [14] drawn from statistical physics [15] and, ultimately, Boltzmann’s 19th-century work [16]. The particular ensemble method explored here for model-guided discovery is called MINE, which stands for Maximally Informative Next Experiment [17].

## Ensemble methods

Ensemble methods were developed in the 19th century by Boltzmann to describe the motion of an ideal gas [16]. In this situation, there is an Avogadro number (A) of particles in a 1 L box, but only three measurements are made: temperature, pressure, and volume. How is the motion of A particles described with 6A degrees of freedom each described with only three measurements?

With so little data, the data did not strongly support just one model. Boltzmann’s solution was not to give up on identifying one best model but rather to make predictions from an ensemble of models [15]. Omics experiments face exactly the same problem [14], but the paucity of data with respect to the complexity of the model is not as severe as in the problem Boltzmann faced. The interest may be in identifying the dynamics of genes and their products in carbon metabolism [14] or the biological clock [18], but genetics dictates that only a limited number of samples at different time points can be made to identify the system, while there are many parameters required to describe the system. For example, the number of measurements at different time points on the biological clock may be on the order of 60 000 measurements (*n*), but there are over 90 000 rate constants and initial conditions (*p*) in the model that must be estimated [19, 20]. Much as for an ideal gas, averaging over the ensemble allows for detailed predictions about complex biological systems, such as the clock.

A simple example is used to illustrate the approach of ensemble methods. The first step is writing down the model specification for the measurements. There are *n* measurements $Y=\big({y}_1,\ldots, {y}_n$) drawn from an unknown distribution parameterized by *p* parameters in $\theta =\left({\theta}_1,\ldots, {\theta}_p\right),$ and some of these parameters are ancillary parameters, such as $\alpha $ below, in which there is less interest. In addition, the variables $U=\big({u}_1,\ldots, {u}_n$) describe the experimental conditions. For example, the list *U* might specify the SNPs used in a GWAS field trial. Then the model specification would take the form:


$$ P\left(Y|\theta, U\right)=C\exp \left(-H\left(Y|\theta, U\right)\right), $$

where *C* is a normalization constant chosen to make the integral over the data *Y* equal to 1. The quantity $H\left(Y|\theta, U\right)$is known as the Hamiltonian. Ideally, the distribution would be observed directly, but, in practice, what is available are sample moments of the data *Y*.

Since the goal is to identify a model $\theta$ supported by the data *Y*, a change of viewpoint is needed. As in the method of maximum likelihood [21], the model specification $P\left(Y|\theta, U\right)$ is viewed as a function of the model parameters $\theta$, and the data *Y* and experimental conditions *U* are taken as fixed:


(1) \begin{equation*} Q\left(\theta |Y,U\right)={\varOmega}^{-1}P\left(Y|\theta, U\right), \end{equation*}

where $\Omega$ is a normalization constant chosen to make the integral over all parameters $\theta$ in the parameter space equal to 1. This normalization constant is only a function of the data Y. The magnitude of the ensemble $Q\left(\theta |Y,U\right)$, or $Q\left(\theta \right)$ for short, is larger when the model $\theta$ is more supported by the data $Y.$It may be useful to think of the ensemble $Q\left(\theta |Y,U\right)$ as a posterior distribution to the model specification $P\left(Y|\theta, U\right)$ with the two functions, $P\left(Y|\theta, U\right)$ and $Q\left(\theta |Y,U\right)$, connected by Bayes theorem [22].

The ensemble $Q\left(\theta |Y,U\right),$ or $Q\left(\theta \right)$ for short, is the collection of models $\theta$ consistent with the available data Y. Model-averaging with respect to the model ensemble $Q\left(\theta \right)$ allows predictions about the system’s behavior. Instead of identifying one model $\theta$, a distribution of models $Q\left(\theta \right)$ is identified. With the number of parameters *p* being vastly greater than the number of data points n, predictions can still be made and tested with respect to averages computed from the ensemble $Q\left(\theta \right)$.

Monte Carlo methods are used to identify the ensemble $Q\left(\theta \right)$[15, 23] because the model specifications are complicated [18, 24]. A simple example will illustrate how this is done. Take the Hamiltonian viewed as a function of $\theta$ as having the following simple form:


\begin{align*}H\left(\theta \right)=\beta \left[-{\theta}^2+\alpha{\theta}^4\big)\right].\end{align*}

**Figure 1:**
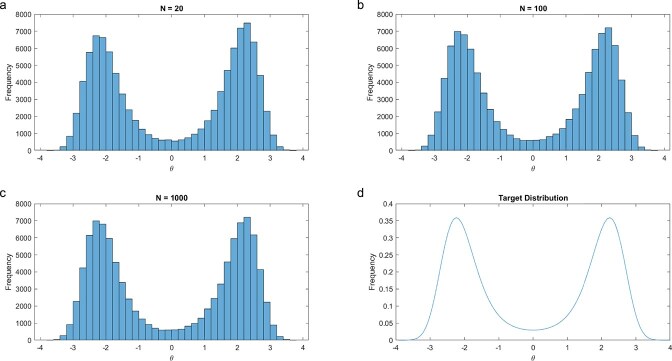
Convergence of an ensemble to the target distribution: (a) ensemble after 20 moves; (b) ensemble after 100 moves; (c) ensemble after 1000 moves; (d) true ensemble or “target distribution.” (d) shows the target distribution as the ensemble converges to the true target distribution under a Monte Carlo experiment (a–c). The starting guess at the parameter $\theta$ was 3. After each of 20, 100, and 1000 moves, 10 000 samples of $\theta$ from the resulting distribution were drawn to characterize the ensemble. The ancillary parameters were $\alpha =0.1$ and $\beta =1.0.$ The plots were created in MATLAB_R2018B (https://www.mathworks.com/products/matlab.html).

The model parameter $\theta$ is the one we are truly interested in, and the remaining parameters $\alpha$ and $\beta$ are ancillary. A graph of the ensemble $Q\left(\theta \right)=\exp \left(-H\left(\theta \right)\right)$ is shown in Fig. 1d. There are two maxima in the ensemble or equivalently, two minima in the Hamiltonian. The goal is to reconstruct the ensemble $Q\left(\theta \right)$ by Monte Carlo for prediction.

In this example, we are in the perfect world in which the ensemble, or equivalently the Hamiltonian, is observed from 10,000 values after each move in the Monte Carlo experiment. To reconstruct the ensemble $Q\left(\theta \right)$by Monte Carlo at each move a new model parameter ${\theta}^{\prime }$ is drawn from the ensemble $Q\left(\theta \right)$when the current proposal is the model parameter $\theta$. The goal is to move into a region of the parameter space that is well supported by the ensemble $Q\left(\theta \right)$ in the equilibration phase. Once equilibrated many 1000 s or 10 000 s of models are accumulated that are well supported to reconstruct the ensemble from the sample histogram of these $\theta$-values [18]. The question remains how to choose the well-supported $\theta$-values.

One greedy approach to moving in the parameter space is to draw a model parameter ${\theta}^{\prime }$ and proceed uphill using some procedure like steepest ascent to climb the hill(s) in the ensemble. As shown in Fig. 1, this might lead to a local maximum. In fact, in Fig. 1, there are two such maxima. To avoid local maxima, a model parameter ${\theta}^{\prime }$ is drawn randomly from the ensemble $Q\left(\theta \right)$, being greedy when there is an improvement in the ensemble probability, i.e. $Q\left({\theta}^{\prime}\right)>Q\left(\theta \right)$ or equivalently $H\left({\theta}^{\prime}\right)<H\left(\theta \right)$, but occasionally when Q$\left({\theta}^{\prime}\right)<Q\left(\theta \right)$, move downhill anyway. The occasional downhill move may allow escape from a local maximum. In practice in systems biology, it may be more appropriate to think of the ensemble surface as gently rolling hills as on a golf course because the data are limited ($n<p\big).$

Metropolis and colleagues [25] developed a stochastic search procedure in statistical physics for this and now many other optimization problems [23]. The probability of a move is:


$$ p=\min \left(1,\frac{Q\left({\theta}^{\prime}\right)}{Q\left(\theta \right)}\right). $$

The probability *p* of a move from $\theta \to{\theta}^{\prime }$ occurs with probability 1 if the proposed move takes us uphill, but if the proposed move takes us downhill, then the probability of a move downhill decreases with the amount of drop from $Q\left(\theta \right)\to Q\left({\theta}^{\prime}\right)$. The sequence of moves is made 10 000 or more times to move into a region of the parameter space well supported by the data during the equilibration phase.

In the equilibration phase, the inferred ensemble converges to the true ensemble known as the target distribution (Fig. 1). The Monte Carlo search for this simple model is successful in the reconstruction in <1000 moves and is surprisingly quick, equilibrating in <20 moves. A video displays the reconstruction process (Video S1). Once equilibration is achieved, another sequence of Monte Carlo moves called the accumulation phase is used to build the target distribution. In this simple example, only 1000 moves are needed to carry the ensemble identification into the accumulation phase.

In practice, a sweep is introduced to describe the number of moves taken to visit each model parameter on average once. A standard equilibration run and an accumulation run is 40 000 sweeps, which will vary in practice with the complexity of the model [18, 19].

## MINE

Once an ensemble method produces a collection of models supported by the data, then it is possible to make predictions from the ensemble distribution about the next experiment. By averaging some variable of interest over the models in the ensemble distribution $Q\left(\theta |Y,U\right)$, a prediction can be made, given the current data *Y* and the experimental conditions *U*. For example, *Y* might be plant biomasses measured in year 1 of a 5-year GWAS experiment to identify SNPs to predict biomass in sorghum with a certain collection of SNPs from the Bioenergy Accession Panel (BAP) [26]. The question is what accessions are to be used in Year 2 to specify design U. In Year 1, 79 accessions are measured in the GWAS and can be used to inform the SNP choice in Year 2.

**Figure 2:**
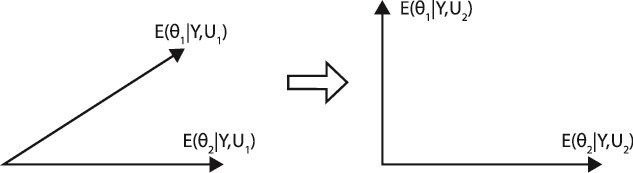
Two models can be better distinguished by their predictions in the next experiment if their predictions are less correlated. The predictions of model ${\theta}_1$ and ${\theta}_2$ under experimental condition *U*_1_ are the expectations ${f}_1=E\left({\theta}_1|Y,{U}_1\right)$ and ${f}_2=E\left({\theta}_2|Y,{U}_1\right)$, respectively. If the two models ${\theta}_1$ and ${\theta}_2$are chosen independently from the model ensemble $Q\left(\theta |Y,U\right)$, the expectations are calculated with respect to the product density $Q\left({\theta}_1|Y,U\right)Q\left({\theta}_2|Y,U\right)$, where *U* = *U*_1_ or *U*_2_.

One way to make this choice is to select experimental conditions permitting us to distinguish the models in the ensemble $Q\left(\theta |Y,U\right)$ identified from Year 1. The best way to distinguish experimentally two models randomly chosen from the model ensemble is if the predictions $F\left(\theta, Y\right)$ of each model ($\theta ={\theta}_1\ or\ {\theta}_2$) are orthogonal as shown in Fig. 2. For experiment 1 on the left, the predictions of the two selected models ${\theta}_1$ and ${\theta}_2$ are correlated and are harder to distinguish under experimental conditions U_1_. The same two models under experimental conditions *U*_2_ are easier to distinguish—model ${\theta}_1$ is easily tested against model ${\theta}_2$. The goal of a MINE criterion is then to support making “the angle” between the two predictions of a random pair in the ensemble as large as possible on average in year 2 as a function of the experimental conditions *U* and current ensemble $Q\left(\theta |Y,U\right)$ identified from the data in Year 1.

There are two standard ways to measure the associations between the predictions [6]. One is by the covariances between the predictions of the data *Y* (MINE by Covariance Ellipsoid Volume); the other is by the correlations between the predictions of the data *Y* (MINE by Correlation Ellipsoid Volume). There are a variety of reasons for advocating the use of MINE by Correlation Ellipsoid Volume [6]. One of the main reasons is that when there are a large number (*p* > > *n*) of almost linearly dependent observations as found in practice, it would be highly desirable to emphasize the new directions in the data *Y* as done by Correlation Ellipsoid Volume. The new directions in *Y* in the next year depend on the choice of design matrix *X*. Denote by E the correlation matrix between the components of *Y*. The MINE Correlation Ellipsoid Volume is then a determinant (det):


$$ V(U)=\det \left(E(U)\right). $$

When the predictions are on average highly correlated (Fig. 2a), the determinant is nearly zero. When the predictions are nearly orthogonal (going in new directions) (Fig. 2b), the determinant is nearly 1.

**Figure 3:**
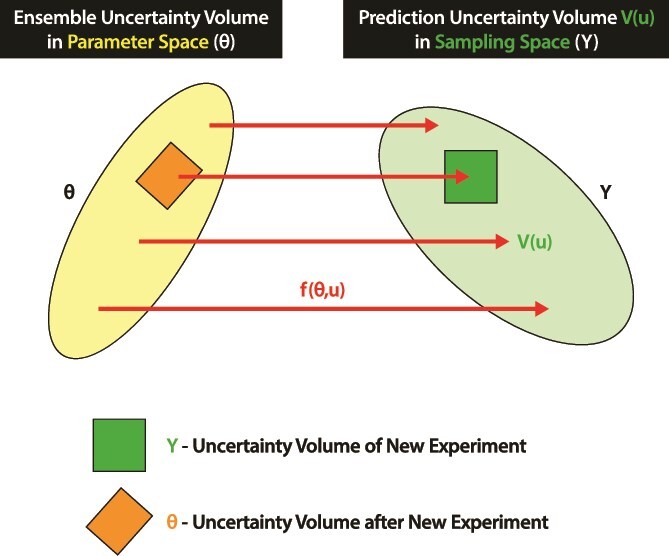
MINE is analogous in function to the optics on a microscope. The data *Y* are the objects in the field of view. The models $\theta$ are in the image. The MINE criterion with the predictions $F\left(\theta, U\right)$ is the optics. The uncertainty volume in the image is the magnification measured by the MINE criterion, *V*(*u*) = det(E(U)). From [44].

A microscope analogy [11] provides insights into how MINE works (Fig. 3). MINE is highly analogous to a microscope and its optics. The object in the microscope field described by the data *Y* is the observed system. MINE, like the optics of the microscope, picks up each component of Y through the prediction $F\left(\theta, Y\right)$ about the system. For example, $F\left(\theta, Y\right)$ could be the list of predictions of plant biomasses in a GWAS study. The optics ($F\left(\theta, Y\right)$) and, likewise, MINE then magnify the predictions to create the image or model of the system (Fig. 3).

The microscope has a field of view of the object, which we refer to as the Uncertainty Volume of the new experiment *Y*. The uncertainty in the observations on the field of view comes from our uncertainty about the optics controlled by $\theta$ and in the measurements *Y* on the object. The optics (predictions) then translate the Uncertainty Volume V(U) in the sample space into an image, the Uncertainty Volume in the parameter space. The result is that an Uncertainty Volume in the sample space (object) is mapped by the optics $F\left(\theta, Y\right)$ to the Uncertainty Volume in the parameter space (image).

The magnification applied to the object is adjusted to reduce the Uncertainty Volume in the parameter space (image). Another interpretation of the image quality is given by the determinant det(E(U)). The determinant is a measure of the volume of a parallelepiped defined by the Uncertainty Volume in the Sample Space [27]. The determinant is also a measure of the Uncertainty volume in the parameter space (inside the ensemble). As the magnification knob is twiddled, the clarity of the image (model parameters) is increased and uncertainty is reduced (Fig. 3). If the parallelepiped is squashed in the parameter space, less details from the observations *Y* in the sample space are being retained in imaging (i.e. model fitting). MINE is doing the focusing and representing the object in higher clarity in the image constructed by the observer using MINE.

### A simple model for predicting hyphal extension colonization by arbuscular mycorrhizal fungi in plant roots to illustrate MINE

Mixture experiments are used in population genetics [28] and science and engineering in general [29]. Mixture experiments are examples of linear models that are the focus of experimental design [2]. In these designs, there is a mixture of treatments in different proportions affecting some dependent variables of interest. Mixture experiments can be used to study how different arbuscular mycorrhizal fungi (AMF) affect the health of the plant through colonization of the root system. The assembly of the AMF biome in plant roots is a product of choices imposed by the plant genotype [30, 31], competition between AMF, ecological drift [9], historical contingency [32], abiotic factors such as phosphorous (P) and nitrogen (N) in the soil [33, 34], and other factors. Consider three AMF species, *S*_1_, *S*_2_, and *S*_3_, competing for colonization area in the plant roots of sorghum [9] of ‘one’ plant genotype. These AMF are potential partners with the plant in one of the oldest symbioses on the planet [35]. Potentially the plant provides carbon, and, in return, potentially, the AMF hyphal network provides P and N, like an extended root system. The success of this partnership is measured in part by AMF hyphal extension in the roots and the resulting biomass of the plant host [36]. To study this symbiosis, the experimenter inoculates sorghum with a mixed population of at least 10% of the coenocyte cells being *S*_1_, at least 15% being *S*_2_, and at least 5% of the coenocyte cells being *S*_3_. The mixed coenocyte inoculum is a coculture in the plant root cells. Denoting respective spore percentages by *u*_1_, *u*_2,_ and *u*_3_, respectively, *u*_1_, *u*_2_, and *u*_3_ are thus constrained by lower limits,


(2) \begin{equation*} u_1\ge u_1^{(lo)}=0.10,u_2\ge u_2^{(lo)}=0.15,u_3\ge u_3^{(lo)}=0.05, \end{equation*}

and by the normalization condition


(3) \begin{equation*} u_1+u_2+u_3=1. \end{equation*}

Given eq. (3), only two of the three species fraction values can be freely chosen. In the following, we will use proportions *u*_1_ and *u*_2_ as those two free variables, with *u*_3_ then being determined *via* eq. (3). Furthermore, the proportions *u*_1_ and *u*_2_ are then subject to upper and lower bounds, resulting from Eqs. (2) and (3). When referring, below, to experimenters freely choosing (*u*_1,_  *u*_2,_  *u*_3_), it should be understood that these choices must be within the constraints imposed by conditions (2) and (3).

Assume that, by setting appropriate experimental conditions, the experimenter can construct an inoculum with a constant total spore population size, *N*_c_, and constant species fractions, *u*_1_, *u*_2,_ and *u*_3_. Assume also that, subject to the foregoing constraints (2) and (3), the experimenter can precisely set the values of *u*_1_, *u*_2_, *u*_3,_ and *N*_c_.

Each of the three AMF taxa can increase its rate of occupancy of the root space in the plant, denoted by ${\theta}_1$, ${\theta}_2$ and ${\theta}_3$, for species, *S*_1_, *S*_2,_ and *S*_3_, respectively. The experimenter wishes to determine, or at least impose constraints on, the values of these rates in percent area increase, ${\theta}_1$, ${\theta}_2$ and ${\theta}_3$, by performing a sequence of time-series experiments wherein the linear filament extension in a root image, denoted by *y*(*t*), is measured as a function of time, *t*, at certain time points,


$$ t_1,t_2,\ldots, t_K. $$

Here, *K* is the total number of experimental observation time points. Each experiment thus produces a series of observed filament extension amounts, *y*(*t_k_*) for *k* = 1,2,…, *K*, denoted by


$$ y_1,y_2,\ldots, y_K. $$

That is, *y_k_* is the value of *y*(*t*) observed at time *t_k_*, with *k* = 1,2, …, *K* labeling the different observation time points. Each of these experiments is to be performed on a spore population begun with a different combination, (*u*_1,_  *u*_2,_  *u*_3_), of AMF inoculation fractions. For simplicity, assume, however, that the values of the rates of hyphal extension, ${\theta}_1$, ${\theta}_2$, and ${\theta}_3$, remain the same throughout all these experiments, i.e. assume that the hyphal extension rates, ${\theta}_1$, ${\theta}_2$, and ${\theta}_3$, do not change when the experimenter changes the population composition (*u*_1,_  *u*_2,_  *u*_3_) from one experiment to the next as in a race tube experiment [6, 37]. For simplicity, we will refer to ${\theta}_1$, ${\theta}_2$, and ${\theta}_3$ as the rates of colonization success.

The extraction of any information about the success rate in root colonization, ${\theta}_1$, ${\theta}_2$, and ${\theta}_3$, from the experimental time series data, *y_k_*, requires, a ‘mathematical model’ that treats the rates ${\theta}_1$, ${\theta}_2$, and ${\theta}_3$, as well as the known ‘experimental control parameters’, *u*_1,_  *u*_2,_ and *u*_3_, as input parameters. The model must then use these input parameters to provide a ‘predicted value’ for each experimental observation, *y_k_*, the hyphal extension colonized in a plant root. For a given experimental data point, *y_k_*, we denote the corresponding value predicted by the model by *f_k_*. Obviously, whatever the model predicts depends on the model input parameters, ${\theta}_1$, ${\theta}_2$, ${\theta}_3$, *u*_1,_  *u*_2_, and *u*_3_, that were used to make the prediction. We will therefore often write *f_k_* as a ‘function’ of these input parameters, i.e. as


$$ fk\left({\theta}_1,{\theta}_2,{\theta}_3,u_1,u_2,u_3\right), $$

to make it explicit that *f_k_* is dependent on the assumed values of the rate parameters ${\theta}_1$, ${\theta}_2$, and ${\theta}_3$, and on the given values of the control parameters, *u*_1,_  *u*_2_, and *u*_3_, set by the experimenter.

For the scenario assumed here, i.e. for a mixed population of spores from three AMF species jointly producing percent root colonization, X, at constant rates per spore cell type, a simple mathematical model for *f_k_* is easy to construct. Assume that the mixed cell population is established, and starts producing colonization, at time $t=0$, with no initial colonization length *X* being present at that time. Then, the total percent colonization X produced by the entire AMF population in the roots, by observation time ${t}_k$, is given by:


(4) \begin{align*}X = f_{k} ( {\theta}_1, {\theta}_2 , {\theta}_3, u_{1}, u_{2}, u_{3}) = {N}_{\mathrm{c}}\, {u}_1\, {\theta}_1 {t}_k + {N}_{\mathrm{c}}\, {u}_2{\theta}_2{t}_k+{N}_{\mathrm{c}}\, {u}_3{\theta}_3\, {t}_k \end{align*}

The percent colonization *X* can be measured in roots by bright field microscopy [38–41].

To understand this linear model, which is linear in the model parameters $\theta,$ recall here that${N}_{\mathrm{c}}$ is the total number of AMF in the inoculum, and hence ${N}_{\mathrm{c}}{u}_1$ is the number of S_1_-cells in the inoculum. Hence, ${N}_{\mathrm{c}}{u}_1\, {\theta}_1$ is the rate of increase by all S_1_-cells combined producing percentage root area X-contribution. Each spore produces a hyphopodium by which to colonize the root cortex. Recall now that


(5) \begin{align*} &\left(\mathrm{Rate}\ \mathrm{of}\ \mathrm{in}\mathrm{crease}\ \mathrm{in}\ \mathrm{colonization}\ \mathrm{length}\right)\times \left(\mathrm{Time}\right)\nonumber\\&\qquad\qquad\qquad\qquad\qquad\ \, \quad=\left(\mathrm{total}\ \mathrm{length}\ \mathrm{colonized}\right)\!. \end{align*}

Hence, the length colonized, by all AMF *S*_1_-cells combined, by time ${t}_k$, is ${N}_{\mathrm{c}}{u}_1\, {\theta}_1\, {t}_k$. Likewise, the length colonized of the roots produced by all AMF *S*_2_-cells and by all *S*_3_-cells, by time ${t}_k$, are ${N}_{\mathrm{c}}{u}_2\, {\theta}_2\, {t}_k$ and ${N}_{\mathrm{c}}{u}_3\, {\theta}_3\, {t}_k$, respectively. We then obtain *f_k_*, i.e. the predicted total amount of hyphal extension colonized X produced by all cells until time ${t}_k$, by simply adding up the foregoing three X-contributions from all three AMF species. The result is eq. (4).

Suppose we have performed multiple experiments, to be labeled by an “experiment index” $\ell =1,2,\ldots L$, where *L* is the total number of experiments. In each experiment, a different AMF species composition (*u*_1,_  *u*_2_, *u*_3_) was used. To distinguish these *u*_1,_  *u*_2,_ and *u*_3_, used in the different experiments, we therefore have to label ‘them’ with the additional index $\ell$, as ${u}_1^{\left(\ell \right)},{u}_2^{\left(\ell \right)},\text{and}\ {u}_3^{\left(\ell \right)}$, for $\ell =1,2,\ldots L$. Consequently, a different time series of X-data, *y*_1_, *y*_2_, …, *y_K_*, was observed in each experiment, and we therefore also have to label the observed data, *y*_1_, *y*_2_, …, *y_K_* with the additional index $\ell$, as ${y}_1^{\left(\ell \right)},{y}_2^{\left(\ell \right)},\ldots, {y}_K^{\left(\ell \right)}$, for $\ell =1,2,\ldots L$. Also assume that each data point, ${y}_k^{\left(\ell \right)}$, has been measured with some experimental uncertainty, quantified by an experimental standard deviation ${\sigma_k}^{\left(\ell \right)}$. The ${\chi}^2$-function (or by another name, the Hamiltonian) is then given by:


(6) \begin{align*} {\chi}^2( \theta , U) =\sum_{\ell =1}^L\sum_{k=1}^K\frac{1}{{\left({\sigma_k}^{\left(\ell \right)}\right)}^2}{\left[{y_k}^{\left(\ell \right)}-{f}_k\left(\theta, {u}^{\left(\ell \right)}\right)\right]}^2. \end{align*}

To simplify and compactify the notation, we have introduced here the following abbreviations:


(7) \begin{equation*} \theta := \left({\theta}_1,{\theta}_2,{\theta}_3\right); \end{equation*}

(8) \begin{equation*} {u}^{\left(\ell \right)}:= \left({u}_1^{\left(\ell \right)},{u}_2^{\left(\ell \right)},{u}_3^{\left(\ell \right)}\right); \text{ for } \ell =1,2,\ldots L; \end{equation*}

(9) \begin{equation*} U:= \left({u}^{(1)},{u}^{(2)},\ldots{u}^{(L)}\right)=\left( {u}_1^{(1)},{u}_2^{(1)},{u}_3^{(1)},{u}_1^{(2)},{u}_2^{(2)},{u}_3^{(2)},\ldots{u}_1^{(L)},{u}_2^{(L)},{u}_3^{(L)}\right) \end{equation*}

That is, $\theta$ (without subscript) is shorthand for a vector that comprises the rates of colonization ${\theta}_1,{\theta}_2$, and ${\theta}_3$. The ${u}^{\left(\ell \right)}$ (without subscript) denotes the vector of the three AMF species inoculation fractions used in experiment number $\ell$, and *U* is the vector comprising the species fractions from ‘all’ experiments combined. Note that $\theta$ does not have an $\left(\ell \right)$-superscript here because ${\theta}_1,{\theta}_2$, and ${\theta}_3$ are assumed to have the same values in all experiments.

Note that ${f}_k\left(\theta, {u}^{\left(\ell \right)}\right)$ is the model prediction of hyphal extension, from eq. (4), for ${y_k}^{\left(\ell \right)}$, i.e. for the *k*th time series data point for percent root area colonized observed in the $\ell$th experiment. The square of the so-called ‘residual’, on the right-hand side of eq. (6),


(10) \begin{equation*} {r}_k^{\left(\ell \right)}\left(\theta, {u}^{\left(\ell \right)}\right):= {y_k}^{\left(\ell \right)}-{f}_k\left(\theta, {u}^{\left(\ell \right)}\right), \end{equation*}

thus measures the deviation of the model prediction ${f}_k\left(\theta, {u}^{\left(\ell \right)}\right)$ from the experimental observation of hyphal extension ${y_k}^{\left(\ell \right)}$: The larger ${\big({r}_k^{\left(\ell \right)}\big)}^2$, the worse, i.e. greater, is the deviation of the model prediction, ${f}_k\left(\theta, {u}^{\left(\ell \right)}\right)$, from the observed data point, ${y_k}^{\left(\ell \right)}$. By taking the sum of all squared residuals, the ${\chi}^2$-function in eq. (6) thus provides a composite measure of the overall deviation of the model predictions from the data, for ‘all’ data points on hyphal length colonized combined. In the least-squares fitting approach, the “best possible” choice of model parameters is then obtained by finding a parameter combination, $\left({\theta}_1,{\theta}_2,{\theta}_3\right)$, which minimizes this deviation, i.e. by minimizing ${\chi}^2$($\theta$, *U*) with respect to ${\theta}_1,{\theta}_2$, and ${\theta}_3.$ In the following, let ${\theta}^{\left(\mathrm{b}\right)}\equiv \left({\theta}_1^{\left(\mathrm{b}\right)},{\theta}_2^{\left(\mathrm{b}\right)},{\theta}_3^{\left(\mathrm{b}\right)}\right)$ denote the best possible parameter combination that minimizes ${\chi}^2$($\theta$, *U*).

Note, in passing, that the squared residuals entering into ${\chi}^2$($\theta$, *U*) in eq. (6) are weighted by the reciprocals of the variances, ${\left({\sigma_k}^{\left(\ell \right)}\right)}^2$. This means that experimental data points with larger experimental uncertainties carry less weight and have less of an effect on the choice of the optimal, “best match” parameter combination, ${\theta}^{\left(\mathrm{b}\right)}$, than data points with smaller experimental uncertainties. In that sense, ${\theta}^{\left(\mathrm{b}\right)}$ can be regarded as a ‘weighted compromise” between all data points, ${y_k}^{\left(\ell \right)}$.

While there are, in principle, many different ways to define an ensemble probability distribution function having these general characteristics, an obvious, simple choice for eq. (1), supported by statistical theory [11], is given by:


(11) \begin{equation*} Q\left(\theta\, |\, U\right)=\frac{1}{\Omega}\, {e}^{-{\chi}^2\left(\theta, U\right)/2} .\end{equation*}

The $1/\Omega$-factor in eq. (11) is a normalization factor, chosen to ensure that the ensemble probability density function or PDF integrates to a probability of 1. That is, for our model for a mixture experiment with $\theta \equiv \left({\theta}_1,{\theta}_2,{\theta}_3\right)$, the $\Omega$ is chosen such that


(12) \begin{equation*} {\int}^{\theta_{\mathrm{Hi}}}_{\theta_{\mathrm{Lo}}}\kern0.5em {\int}_{\theta_{\mathrm{Lo}}}^{\theta_{\mathrm{Hi}}}\kern0.5em {\int}_{\theta_{\mathrm{Lo}}}^{\theta_{\mathrm{Hi}}}\, Q\left({\theta}_1,{\theta}_2,{\theta}_3\, |\, U\right)\, {d\theta}_1\, {d}{\theta}_2\, {d}{\theta}_3=1. \end{equation*}

Here, ${\theta}_{\mathrm{Lo}}$ and ${\theta}_{\mathrm{Hi}}$ denote, respectively, a reasonable lower and upper limit imposed on ${\theta}_1,{\theta}_2$, and ${\theta}_3$. Eq. (11) is then to be understood to hold only when ${\theta}_1,{\theta}_2$, and ${\theta}_3$ each falls within the interval between ${\theta}_{\mathrm{Lo}}$ and ${\theta}_{\mathrm{Hi}}$; if ${\theta}_1$, ${\theta}_2$, or ${\theta}_3$ lies outside of this interval, we set $Q\left(\theta\, |\, U\right)=0.$

Notice that $Q\left(\theta\, |\, U\right)$ in eq. (11) has the desired general characteristics: For very large values of ${\chi}^2\left(\theta, U\right)$, the exponential function ${e}^{-{\chi}^2\left(\theta, U\right)/2}$, and hence $Q\left(\theta\, |\, U\right),$ becomes very small; for smaller values of ${\chi}^2\left(\theta, U\right)$, $Q\left(\theta\, |\, U\right)$ becomes larger. Hence, $\theta$-choices whose model predictions agree poorly with the experimental data will have a low probability of being drawn from $Q\left(\theta\, |\, U\right)$; $\theta$-choices whose model predictions agree well with the experimental data will have a higher probability of being drawn from $Q\left(\theta\, |\, U\right)$.

Given $Q\left(\theta\, |\, U\right)$, we can now calculate, for example, expectation values, variances, and histograms of any observable quantity, $A\left(\theta \right)$, which the model allows us to predict as a function of $\theta$. Specifically for the expectation value, $\text{E}\left[\ldots \right]$, and variance, ${\mathrm{\sigma}}^2\left[\ldots \right]$, of such an “observable” $A\left(\theta \right)$, we need to calculate:


(13) \begin{equation*} \text{E}\left[A(.)\right]={\int}_{\theta_{\mathrm{Lo}}}^{\theta_{\mathrm{Hi}}}\kern0.5em {\int}_{\theta_{\mathrm{Lo}}}^{\theta_{\mathrm{Hi}}}\kern0.5em {\int}_{\theta_{\mathrm{Lo}}}^{\theta_{\mathrm{Hi}}}\, A\left(\theta \right)\, Q\left(\theta\, |\, U\right)\, {d}^3\theta, \end{equation*}

with $\theta \equiv \left( {\theta}_1,{\theta}_2,{\theta}_3\right)$ and ${d}^3\theta \equiv \text{d}{\theta}_1\, \text{d}{\theta}_2\, \text{d}{\theta}_3$ for short, and then


(14) \begin{equation*} {\mathrm{\sigma}}^2\left[A(.)\right]=\text{E}\left[{\left(A(.)\right)}^2\right]-{\left(\text{E}\left[A(.)\right]\right)}^2. \end{equation*}

Here, $\text{E}\left[{\left(A(.)\right)}^2\right]$ is obtained, analogous to $\text{E}\left[A(.)\right]$, with $A\left(\theta \right)$ in eq. (13) replaced by ${\left(A\left(\theta \right)\right)}^2$.

Within the ensemble approach, $\mathrm{E}\left[A(.)\right]$ can serve as a prediction of a representative value of $A\left(\theta \right)$, given the experimental control parameters *U* and prior experimental data, ${y_k}^{\left(\ell \right)}$ for all $\ell$ and all *k*. However, the ensemble approach also allows us to evaluate the ‘uncertainty’ of that prediction, by way of $\mathrm{\sigma} \left[A(.)\right]$. Furthermore, with similar expectation value calculations, we can also analyze in more detail the random distribution of $A\left(\theta \right)$ by way of histograms of all possible *A*-values. This would tell us, for example, if the values of $A\left(\theta \right)$ have a uni- or a multimodal distribution, for random $\theta$s drawn from the ensemble $Q\left(\theta \right)$.

These are just a few examples of what kinds of data analyses and model predictions the ensemble approach itself allows us to implement. In the context of the MINE approach of experiment design, we will have to evaluate certain correlations between pairs of observables, *A*$\left(\theta \right)$ and *B*$\left(\theta \right)$, say. This will require the calculation of expectation values of the general form $\mathrm{E}\left[A(.)\, B(.)\right]$, with $A\left(\theta \right)$ in eq. (13) replaced by the product $A\left(\theta \right)B\left(\theta \right)$.

**Figure 4:**
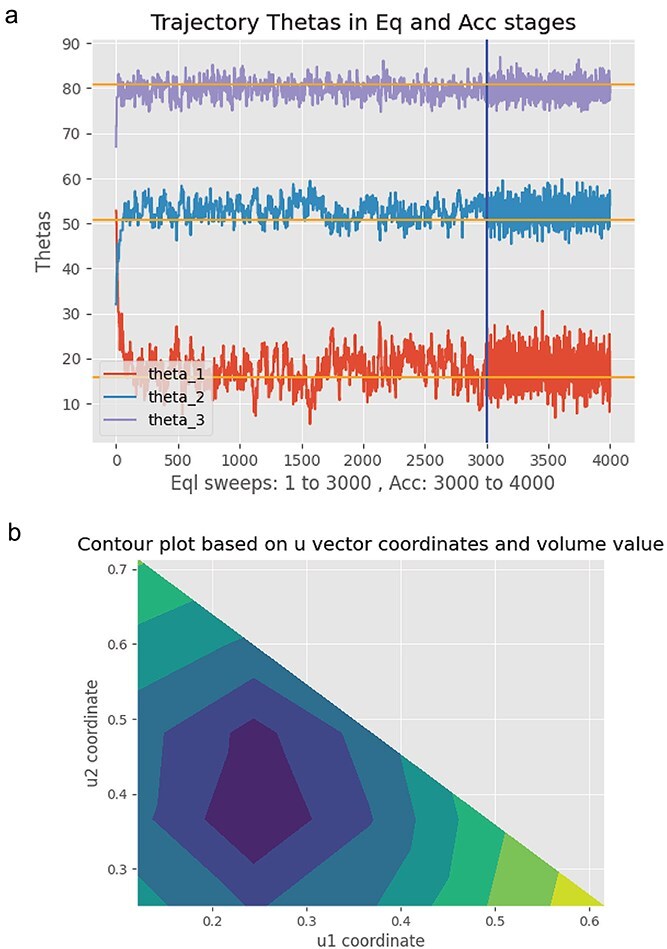
An ensemble method to identify the mixture experiment’s hyphal extension rates $\theta$ is carried out on simulated data from the mixture experiment, and MINE is used to choose the next mixture experiment with inoculation proportions *u*_1_ and *u*_2._ (a) illustrates the ensemble method on simulated data for a mixture experiment of AMF colonizers. The orange lines are the true colonization rates $\theta$. In the Monte Carlo experiment, the estimated rates are plotted as a function of sweep, a visit on average once to each of the three rates $\theta .$In the first 3000 sweeps, the Monte Carlo experiment is equilibrated to get in the neighborhood of parameters $\theta$ that fit the simulated data. In the accumulation phase (last 1000 sweeps) the estimates of $\theta$ are accumulated to form the ensemble estimate. (b) presents the next MINE mixture experiment recommended. The contour plot is of the MINE criterion det(E) as a function of the mixture of spore inoculum proportions *u*_1_ and *u*_2._

The evaluation of all the foregoing expectation values usually requires numerical techniques to carry out the $\theta$-integrations as in eq. (13). In general, the $\theta$-space is very high-dimensional, far greater than the $\theta$-dimension of $\text{dim}\, \left(\theta \right)$=3 in our simple model here. Markov chain Monte Carlo methods are then the only approach available to perform the required expectation value calculations efficiently for omics experiments and field studies [15] (see Ensemble Methods section). In Fig. 4a is a simulation of the mixture experiment with the application of the ensemble method to the simulated data. The ensemble method converges quite well to the true colonization rates $\theta$.

Assume *L* prior experiments have already been performed, with experimental control parameter vectors ${u}^{\left(\ell \right)}$, as defined in eq. (8), and observed values ${y_k}^{\left(\ell \right)}$, for $\ell =1,2,\ldots L$ and $k=1,2,\ldots K.$ The experimental data points, ${y_k}^{\left(\ell \right)}$, combined with the corresponding model predictions, ${f}_k\left(\theta, {u}^{\left(\ell \right)}\right),$ from eq. (4), define an ensemble PDF, $Q\left(\theta |\, U\right)$, *via* eqs. (6) and (13). The $Q\left(\theta |\, U\right)$, in turn, will determine $V(U),$ the predicted uncertain volume of the observables, to be measured in the new experiment(s), as follows:

As the simplest case, assume that we want to design just ‘one’ new experiment, with a new experimental control parameter vector $u\equiv \left( {u}_1,{u}_2,{u}_3\right)$. The MINE objective is then to choose to input inoculation proportions $u$ so as to maximize the information content of the new experiment about the rates of production of colonization by hyphal extension (*X*), by maximizing the predicted uncertainty volume of the observables to be measured. In our simple mixture experiment example, there are *K* such observables in any experiment: the hyphal extension *X*-amounts to be measured at times *t_k_*, for $k=1,2,\ldots K$. The predicted values for these observed hyphal extensions *X* are then ${f}_k\left(\theta, u\right)$, as defined by the model eq. (4), for given $\theta$ and $u$. These predicted values for these *K* observations can be thought of as the components of a vector in a *K*-dimensional space, the so-called ‘observation space’ (Fig. 3). For a given $\theta$ and $u$, this vector of ‘predicted observations’ is in the following denoted by $f\left(\theta, u\right)$, and given by


(15) \begin{equation*} f\left(\theta, u\right):= \left({f}_1\left(\theta, u\right),{f}_2\left(\theta, u\right),\ldots{f}_K\left(\theta, u\right) \right). \end{equation*}

We can now use the ensemble PDF, $Q\left(\theta, U\right)$, to define, in some way, a volume of likely $\theta$s in $\theta$-space. If we let $\theta$ sweep over that finite volume then, by eq. (15), $f\left(\theta, u\right)$ will sweep over some corresponding finite volume (or hyper-surface) in the observation space: the ‘uncertainty volume’ of the predicted observation vector, to be denoted by $V(u)$, for given $u$, and illustrated in Fig. 3.

There is, of course, no precise prescription of how to define a volume of likely-$\theta$ in $\theta$-space, or a corresponding uncertainty volume, $V(u)$, in observation space. That definition is not unique: it requires some arbitrary but reasonable choices to be made. In the following, two specific possible choices for $V(u)$ will be discussed.

### MINE by Covariance Ellipsoid Volume

In the covariance matrix approach, we define $ V(u) $ in terms of the uncertainty ellipsoid, constructed from the covariances of the *K* observable predictions, $ {f}_1\left(\theta, u\right),{f}_2\left(\theta, u\right),\ldots{f}_K \left(\theta, u\right) $, subject to the ensemble PDF $ Q\left( \theta, U\right) $. Let $ {D}_{kj}(u) $ denote those covariance matrix elements, i.e. for $ k,j=1,2,\ldots K $, let


(16) \begin{equation*} {D}_{kj}(u):= \mathrm{E}\left[{f}_k\left(.,u\right)\, {f}_j\left(.,u\right)\, \right]-\mathrm{E}\left[{f}_k\left(.,u\right)\right]\, \mathrm{E}\left[{f}_j\left(.,u\right)\, \right], \end{equation*}

with expectation values E[…] defined as in eq. (13). On general mathematical grounds, the corresponding $K\times K$ covariance matrix, $D(u),$ is symmetric and positive semidefinite. Therefore, *D* has *K* real, non-negative eigenvalues, ${\lambda}_{\nu }$; and it has an orthonormal basis of corresponding *K*-dimensional eigenvectors, ${e}^{\left(\nu \right)},$ with $\nu =1,2,\ldots K$. That is, for $k,j,\nu, \mu =1,2,\ldots K$, we have:


(17) \begin{equation*} \sum_{j=1}^K\, {D}_{kj}(u)\, {e}_j^{\left(\nu \right)}={\lambda}_{\nu }\, {e}_k^{\left(\nu \right)} \end{equation*}

(18) \begin{equation*} {\lambda}_{\nu}\ge 0 \end{equation*}

(19) \begin{equation*} {\sum}_{j=1}^K\, {e}_j^{\left(\nu \right)}\, {e}_j^{\left(\mu \right)}={\delta}_{\nu, \mu } \end{equation*}

(20) \begin{equation*} {\sum}_{\nu = 1}^{K}\, {e}_{k}^{\left( \nu \right)}\, {e}_{j}^{\left( \nu \right)} = \delta_{k,j}. \end{equation*}

The eigenvalues and eigen vectors are, of course, dependent upon *u*, but, for notational simplicity, we have suppressed that functional dependence, i.e. ${\lambda}_{\nu }(u)$ and ${e}_j^{\left(\nu \right)}(u),$ in eqs. (17–20).

By eq. (19), the eigenvectors, ${e}^{\left(\nu \right)}$, are orthogonal, i.e. pairwise perpendicular to each other. The eigenvalues, ${\lambda}_{\nu }$, are the variances of the predicted observation vector, $f\left(\theta, u\right),$along the corresponding eigenvector directions. That is, if we take the projection of the vector $f\left(\theta, u\right)$ onto ${e}^{\left(\nu \right)}(u),$ i.e. let ${p}^{\left(\nu \right)}\left(\theta, u\right)$ denote that projection, with


(21) \begin{equation*} {p}^{\left(\nu \right)}\left(\theta, u\right):= {e}^{\left(\nu \right)}(u)\cdotp f\left(\theta, u\right)={\sum}_{k=1}^K\kern0.50em {e}_k^{\left(\nu \right)}(u)\, {f}_k\left(\theta, u\right), \end{equation*}

then ${\lambda}_{\nu }$ is the variance of that projected $f\left(\theta, u\right)$–vector:


(22) \begin{equation*} {\mathrm{\sigma}}^2\left[{p}^{\left(\nu \right)}\left(.,u\right)\right]={\lambda}_{\nu }, \end{equation*}

where ${\sigma}^2\left[\ldots \right]$ is defined as in eq. (14).

The eigenvalues and eigenvectors of $D(u)$define the so-called “covariance ellipsoid” or “error ellipsoid” of the predicted observation vector, $f\left(\theta, u\right)$, in the *K*-dimensional observation space: The eigenvectors, ${e}^{\left(\nu \right)}$, can be thought of as the orientations of the principal axes of the ellipsoid; the standard deviations of the projections ${p}^{\left(\nu \right)}\left(\theta, u\right),$ i.e. $\mathrm{\sigma} \left[{p}^{\left(\nu \right)}\left(.,u\right)\right]={\sqrt{\lambda}}_{\nu },$ are the lengths of the principal semi-axes along the ${e}^{\left(\nu \right)}$- direction. This ellipsoid serves as our uncertainty volume, and $V(u)$ is given by the product of the semi-axis lengths,


(23) \begin{equation*} V(u)={C}_K\, \sqrt{\lambda_1(u)\, {\lambda}_2(u)\ldots{\lambda}_K(u)}, \end{equation*}

where ${C}_K$ is an unimportant geometrical prefactor,


(24) \begin{equation*} {C}_K=\frac{2{\pi}^{n/2}}{n\, \Gamma \left(\frac{n}{2}\right)}, \end{equation*}

with $\Gamma (x)$ denoting Euler’s gamma function. Eq. (23) can also be written in terms of the determinant of the *D*-matrix:


(25) \begin{equation*} V(u)={C}_K\, \sqrt{\det \left(D(u)\right)}. \end{equation*}

### MINE by Correlation Ellipsoid Volume

In the correlation matrix approach, we define $V(u)$ in terms of an uncertainty ellipsoid constructed from the Pearson correlations of the *K* observable predictions, ${f}_1\left(\theta, u\right),{f}_2\left(\theta, u\right),\ldots\, {f}_K\left(\theta, u\right)$, subject to the ensemble PDF $Q\left(\theta, U\right)$. The Pearson correlation matrix elements, denoted by ${E}_{kj}(u)$, are related to the covariance matrix elements, ${D}_{kj}(u)$, from eq. (16), by


(26) \begin{equation*} {E}_{kj}(u):= \frac{D_{kj}(u)}{\sqrt{D_{kk}(u)\, {D}_{jj}(u)}}. \end{equation*}

Note that ${E}_{kj}(u)$ can also be written as the covariance matrix of the predicted observations, ${f}_k\left(\theta, u\right)$, normalized by their standard deviations:


(27) \begin{equation*} {E}_{kj}(u):= \mathrm{E}\left[{g}_k\left(.,u\right) {g}_j\left(.,u\right) \right]-\mathrm{E}\left[{g}_k\left(.,u\right) \right]\, \mathrm{E}\left[{g}_j\left(.,u\right) \right], \end{equation*}

where


(28) \begin{equation*} {g}_k\left(\theta, u\right):= \frac{1}{\sigma \left[{f}_k\left(.,u\right)\right]}\, {f}_k\left(\theta, u\right) . \end{equation*}

Therefore, the correlation matrix *E* has the same mathematical properties of symmetry and semipositivity as the covariance matrix *D*. Analogous to the covariance ellipsoid constructed from *D*, we can therefore construct a correlation ellipsoid from the eigenvalues and orthonormal eigenvectors of the correlation matrix *E*. Using the volume of the correlation ellipsoid as the uncertainty volume of the predicted observables, we then have, analogous to eq. (23),


(29) \begin{equation*} V(u)={C}_K\, \sqrt{\kappa_1(u)\, {\kappa}_2(u)\ldots{\kappa}_K(u)}, \end{equation*}

where ${\kappa}_1(u),{\kappa}_2(u),\ldots{\kappa}_K(u)$ are the eigenvalues of the correlation matrix *E*. Analogous to eq. (25), we can also write this as


(30) \begin{equation*} V(u)={C}_K\, \sqrt{\det \left(E(u)\right)}. \end{equation*}

The advantages of the volume for the correlation ellipsoid are several. One, *V*(*u*) is a measure of linear dependence of the observables, and the greatest gain in information from an experiment is likely to come from structuring an experiment to increase this linear independence of the observables. In fact, *V*(*u*) is 1 when the observables are linearly independent and is 0 when there is some linear dependence in the observables. Two, *V*(*u*) has well-known statistical properties when the ensemble takes the form of eq. (11) [42].

The surface of the MINE criterion in a contour plot is shown for the next mixture experiment (Fig. 4b). The MINE experiment involves using ~0.25 of AMF1 in the inoculum and ~0.40 of AMF2 in the inoculum to characterize the rates of hyphal extension in the next experiment. As a final note, some theorems about the properties of MINE have been established for the class of linear models, such as the mixture experiments [11]. Code for the ensemble methods is available on GitHub [43, 44].

## Application of MINE to RNA profiling experiments to discover the mechanism of biological clocks

**Figure 5:**
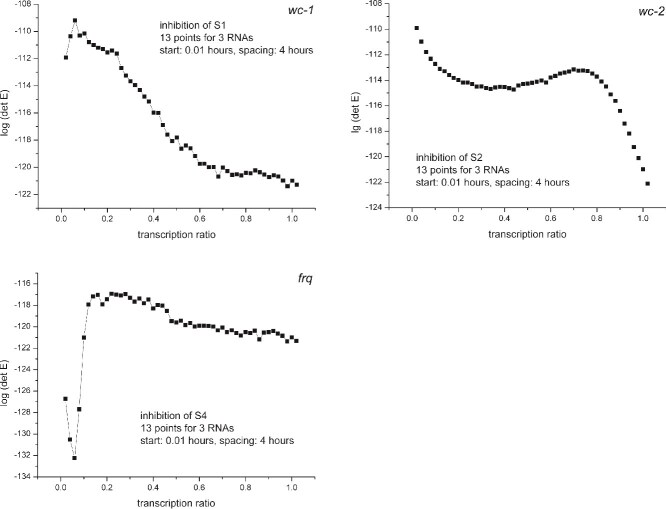
The MINE experiment is a 90% knockdown of the *wc-1* gene. The MINE criterion displayed is the correlation ellipsoid volume det(E(U)), which is graphed as a function of the remaining activity of the three clock mechanism genes. The predictions F are of the log base 10 concentrations over time of *frq*, *wc-1*, and *wc-2* mRNAs over time from the RNA profiling experiments. The mRNA levels were measured at 14 time points over an 8-h window. The drawing is taken from [6].

MINE was developed specifically for this kind of transcriptomics problem and used to close the loop in the computing life cycle (Fig. 5) proposed by Hood and Abersold [45]. Here, the application was to transcriptomic experiments to discover the mechanism underlying circadian rhythms, one of the central problems of systems biology [46]. Transcriptomic experiments have a limited number of time points *n* but have many 1000 s of genes [and hence parameters (*p*)] to be identified in the process [6]. While both MINE criteria using the Covariance by Ellipsoid Volume and Correlation by Ellipsoid Volume were developed, only the Correlation by Ellipsoid Volume was utilized in the end in designing the experiments [6].

Transcriptomics was to be used to explore the mechanism of the biological clock in one of the most well-studied model systems [47], the filamentous fungus, *Neurospora crassa*. Three major components of the clock mechanism were: (i) *frequency* (*frq)*, the gene encoding the oscillator of the system, and a negative regulator [48]; (ii) *white-collar-1* (*wc-1*), the gene encoding the light response element and a positive transcriptional activator for the system [49]; and (iii) and *white-collar − 2*(*wc-2*), a second positive transcriptional activator for the system. Together *wc-1* and *wc-2* encode WC-1 and WC-2 proteins that act as positive elements in the clock through the dimeric complex WCC=WC-1/WC-2, while *frq* encodes a protein FRQ, which acts as the negative regulator for the system. The FRQ protein provides negative feedback to *wc-1* and *wc-2*. All three of these elements appear in a single copy in the *N. crassa* genome, but they have homologs in fly and mammalian systems [49, 50].

Now consider a series of RNA profiling experiments that were conducted, guided by MINE to choose an informative sequence of experiments. The last in a series of three adaptive experiments guided by MINE involved a choice of whether or not to do a knockdown or overexpression experiment on: (i) *frq*; (ii) *wc-1*; or (iii) *wc-2*. The conventional wisdom was to mutate the oscillator gene *frq* [51].

The first step in the MINE application is to make predictions for various mutations in the clock mechanism genes using an available ensemble. The RNA profiles of all 11 000 genes were measured at each of 14 time points. A subset of these RNA measurements included a total of 14 time points on each of the three clock genes so that the prediction vector F had 42 components. Unlike previous models so far considered, the clock model is a nonlinear model (in the parameters describing the model), which specifies a genetic network of nonlinear ordinary differential equations describing the time course of the genes, their cognate RNAs, and proteins [18]. The model ensemble for this clock network was used to predict RNA profiles of *frq*, *wc-1*, and *wc-2* and their correlations under different possible experiments as shown in Fig. 2.

With the correlation matrix in hand for the predictions, the MINE criterion based on the correlation volume ellipsoid in eq. (30) was calculated as a function of the degree of knockdown of the three clock genes (Fig. 5). The result was surprising. A knockdown of *wc-1* was selected as the MINE experiment and used to identify 2323 genes responding to the knockdown [6]. The second surprise from this model-guided discovery process was that ribosome biogenesis was under clock control. This was later confirmed in mammalian systems [52].

A better way to do this MINE calculation would have been to use the transcriptomic data on all 11 000 *N. crassa* genes instead of just the three clock genes driving the system. The ensemble methods now exist for the whole genome-scale network with all of its 1000 s of genes and ensembles now exist for the entire clock network [19, 20, 53]. Using graphical processing unit (GPU) ensemble methods, such as MINE, can now be implemented on a genomic scale with an unknown network structure [19, 53].

## Application of MINE to genome-wide association study field studies for AMF/sorghum project

Consider a GWAS study underway to understand the genetic basis of biomass and AMF colonization in *Sorghum bicolor* using the BAP Panel [26] of 343 plant accessions of varying biomass. There are 232 303 SNPs to characterize each member of the panel after filtering for minor allele frequencies [54]. The focus is on dry weight as a measure of biomass, and AMF colonization is measured using a convolution neural network from imaging AMF in roots [41, 55]. At Time 0, dry weight data were available on each accession in the panel to construct an ensemble [26]. The GWAS study has been running for 5 years [43, 44], and, each year, MINE is being used to select 79 BAP plant accessions for study. The 79 plant seedlings will be assigned randomly to 79 rows with each row consisting of 9 seedlings of identical genotype. The 79 × 9 block was replicated three times in the field. The goal is to discover a relation between plant biomass as a function of SNPs.

**Figure 6:**
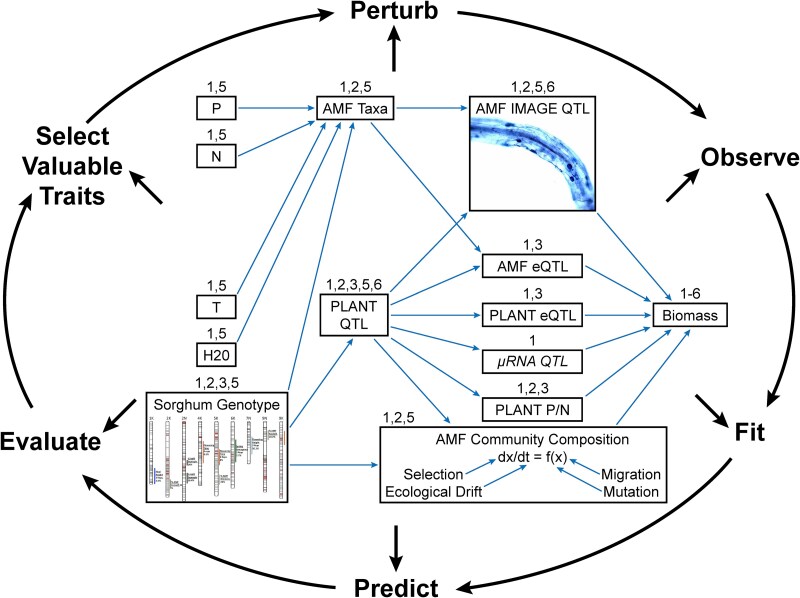
A sequence of MINE experiments is being used in a 5-year GWAS experiment to examine the relation between biomass and SNPs in *S. bicolor* using the BAP accessions [26]. MINE is used to select the BAP accessions to be used each year in order to map AMF colonization and biomass to the sorghum genetic map in a GWAS study. Multiscale structural equation model (SEM) for the project (center boxes and arrows). Lotka–Volterra community models are nested within the SEM and predict associations that affect biomass. The dependent variable is biomass, and the arrows in the diagram denote causal relationships between independent variables in the SEM. In this model, sorghum genotype is the primary independent variable that correlates with the remaining variables. This conceptual model will evolve continuously using the model-guided discovery process of maximally informative next experiment (MINE; outer ring) [6].

In the field study, there are actually a number of plant features that are being measured during the sequence of MINE experiments [44]. These include plant genotype, plant expression Quantitative Trait Loci or eQTLs, the microbiome, tissue total phosphorus (P), nitrogen (N), time of harvest, and other variables relevant to plant health as measured by biomass (Fig. 6). These variables used to predict biomass (as well as AMF colonization and AMF community composition) are held together in a causal diagram representing a structural equation model [56]. The standard model for GWAS experiments is the mixed linear model, which is a special case of the structural equation model in which some of the independent variables are random with mean 0 [57]. For purposes of illustration, a mixed linear model is presented below for an adaptive GWAS experiment underway at Wellbrook Farm, Athens, GA.

There are two sets of measured inputs to the GWAS experiment in Year 1 at Wellbrook Farm, Athens, GA: (i) fixed variables in the design matrix *X*, such as block number, harvest time of each plant, N level, P level; (ii) random intercepts and slope effects *Z* for the genotype of each BAP accession on the other hand. The additive genetic variation in plant genotype as it affects the dependent variable of log dry weight (e.g. biomass) was captured by binning the number of alleles in a given genomic region different from the reference genome using the sum method [58]. The number of SNPs in each chromosomal region was adjusted to ensure that at least each genomic region was 50 kb in size. Since linkage disequilibrium is reported between markers separated by 3.5–35 kb [59], the 50 kb size was chosen to reduce linkage disequilibrium between bins. The result was that the number of bins was 2748 in the 750 Mb sorghum genome with each bin typically having 10–12 genes in sorghum. The number of such alleles in a bin is treated as a continuous random variable with mean 0 and variance component ${\sigma}_i^2$ for the *i*th accession, and summed over regions to obtain a random effect for an accession. The fact that each random effect is the sum of 2748 small random effects of chromosomal regions makes it plausible that the random effect of accession is normally distributed. The fixed effects of block number and BAP genotype, for example, are denoted by the vector $\beta$ and the random effects, by *u*.

These fixed and random effects are used to predict some measure of biomass, log dry weight. In the experiment, there are a total of *n* ~ 606 plants (2–3 plants × 3 blocks × 79 rows) in the field to infer the fixed and random effects, which is less than *p* = 2748 fixed effects +79 variance components. The measurements to be predicted are summarized in the *n* × 1 vector *Y*. The last component of the model is the error in biomass, denoted by ${\epsilon}_i$ for the ith plant in the experiment. The biomass measurements are summarized in an *n* × 1 column vector *Y*.

In Year 1 of the adaptive GWAS experiment, there was no evidence of block effects, and N and P applied were not varied. A total of 79 BAP accessions were planted in a randomized complete block design with 3 blocks, 79 plots in each block, 1 genotype randomly assigned per plot (i.e. a row), and 12 replicates per plot. The mixed linear model for this experiment is reduced to:


(31) \begin{equation*} Y= X\beta + Zu+\epsilon, \end{equation*}

where *Y* is a *n* × 1 vector of observations on biomass. The *n* × *p* matrix *X* is the values of the *p* independent variables (describing each bin) with n observations on each of the chromosomal bins. The *p* × 1 vector $\beta$ are the fixed effects of each chromosomal region. The *n* × *r* matrix *Z* is the *r* = 2748 normalized number of alleles in each accession on n plants in the field. The *r* × 1 vector $u$ is a vector of random effects of each accession on biomass. The errors in the dependent variable, biomass *Y*, are captured in the *n* × 1 vector $\epsilon$. Three of the assumptions of the model are that: (i) the random effects u are independent of the biomass errors $\epsilon$; (ii) the errors $\epsilon$ are normally distributed with mean 0 and variance ${\sigma}^2$; (iii) the random effects u are normally distributed with mean 0 and variance ${\sigma}_{j(i)}^2$ plant I with accession *j*(*i*). That is, the assumptions are that the random effects $u$ and errors $\epsilon$ are independent and normally distributed with mean 0 and variance–covariance matrix $I{\sigma}_i^2$and I${\sigma}^2$, respectively. In the application of eq. (31) taking *Z* = *X* implies that both intercepts and slopes are random as in [41].

Under this model, the prediction of biomass is:


$$ E(Y)= X\beta . $$

The variance components and heritability are used to calculate the variance–covariance matrix *V* of the biomass measurements *Y*:


$$ V\equiv VAR(Y)=\sum_{i=1}^n{Z}_i{Z}_i^{\prime }{\sigma}_{j(i)}^2+{\sigma}^2I=\sum_{i=1}^n{X}_i{X}_i^{\prime }{\sigma}_{j(i)}^2+{\sigma}^2I, $$

where $Z={\left[{Z}_1,\ldots, {Z}_n\right]}^{\prime }={\left[{X}_1,\ldots, {X}_n\right]}^{\prime }$. That is, ${Z}_i$ and ${X}_i$ are the ith row vectors of Z and X, respectively. Each observation ${Y}_i$ has such a 1 × *n* row vector ${X}_i$ to describe the genetics of its accession. Each term ${X}_i{X}_i^{\prime }{\sigma}_i^2$ is an *n* × *n* block. The variance–covariance matrix is diagonal with *p* blocks each with the same diagonal elements ${\sigma}_{j(i)}^2$. The index *j*(*i*) is a lookup that returns the variance component of the *i*th observation as determined from accession *j*. Plant *I* has an assigned accession *j*.

With the assumptions above for the mixed linear model, the ensemble *Q* can be written down as multivariate normal with the $\theta$-vector consisting of the fixed effects $\beta$ and the variance components:


$$ Q\left(\theta \right)=\frac{e^{-\frac{1}{2}\left(Y- XB\right)\prime{V}^{-1}\left(Y- XB\right)}}{{\left(2\pi \right)}^{n/2}{\left|V\right|}^{1/2}} $$

In the first use of MINE in a field experiment, no fertilizers were applied to the field in 2021 at Wellbrook Farm, Athens, GA [44]. The model was reduced to a fixed effects model with the number of alleles in a bin as the set of independent variables using the sum method [58].

The ensemble method was used to fit the models to the published log dry weight data from 3 years from 2013 to 2015 averaged in Florence, SC [26], to make predictions in the use of MINE [44]. Typically, in an omics experiment, there are prior published data available, and this should be used when available [6] to initialize the MINE sequence. A total of 1000 equilibration sweeps were done, and then 1000 sets of model parameters were accumulated, each model parameter set separated by 100 decorrelation sweeps. The chi-square per data point was 6.12 with *n* = 606 dry weight measurements. As a control, the ensemble run was repeated with the only change being 1000 decorrelation steps. The fitted ensemble from all the data simultaneously provides a unified framework for feature selection to avoid overfitting [43, 44].

**Figure 7:**
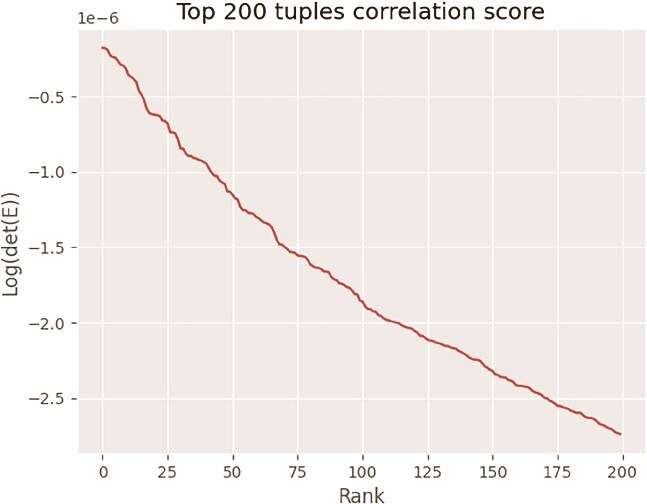
The MINE criterion log(det(E)) was used to select 80 accessions for use in a GWAS experiment at Wellbrook Farm, GA, in 2022. The top 200 selected triples of accessions are ranked by det(D). From these top 200 triples, 80 distinct accessions were selected.

MINE was then applied to the fitted ensemble from Florence, SC, to select 80 accessions for use in 2022 for planting at Wellbrook Farm [44]. The MINE method used was the covariance ellipsoid in eq. (25). The MINE criterion *V*(*u*) conceptually involved evaluating det(D) over all possible $\left(\genfrac{}{}{0pt}{}{343}{80}\right)$ samples from the BAP panel, which is computationally intractable. Instead as an approximation, the MINE criterion was optimized by evaluating det(D) on all $\left(\genfrac{}{}{0pt}{}{343}{3}\right)$ triples drawn from 343 BAP accessions deposited at USDA GRIN in Griffin, GA. The result is shown graphically (Fig. 7). Details of calculating det(D) in the MINE section begin with calculating the covariance matrix of the predictions for next year’s experiment with eq. (16). The eigenvalues are calculated for the covariance matrix using eqs. (17–20). The eigenvalues, in turn, determine det(D) in eq. (23). Code for the ensemble methods and the MINE calculations is available on GitHub [44].

A detailed comparative analysis of MINE in GWAS with classic design approaches is available [43, 44]. The use of MINE in a series of smaller three yearly adaptive experiments is compared directly with a large classic design on the BAP for log dry weight [26]. In this example, the MINE experiments made the GWAS feasible with ~8 project participants per year in the field experiment where a larger classic design using all BAP accessions in 1 year involved over 20 project participants.

As a final note, in the first pass to developing the ensemble methods, such as MINE, for GWAS, the random effects were assigned to accessions rather than individual genes to track the replicates across blocks in the field design each year and to keep the model simpler by limiting the number of variance components to the number of accessions rather than the number of bins. The hypothesis to be tested was simply to ascertain whether or not BAP accessions had an effect on AMF colonization. Future work will include more variance components for each chromosomal bin to fit these more complicated ensembles of models [43].

## Conclusion

There are other potential domains of application of MINE. One example is computer vision models for large image data sets, such as the 15 Terabyte (Tb) data set of plant root images with AMF structures in the root cells recently reported [60]. As such data sets grow, there are choices of structural categories to annotate for ‘balance’ to build a good classification and segmentation model. Then, the ratios of training, validation, and testing sets need to be selected as design parameters, the usual ratios being 8:1:1. Once a criterion for information is decided upon, such as accuracy of classification or F1 or precision as well as for segmentation [i.e. Intersection over Union (IoU)] [41], then MINE could be applied. Classification problems are potentially approachable by MINE in the same way as MINE has been applied to phylogenetic trees in taxon sampling [61, 62]. The goal would be to select promising categories for further annotation to improve classification and segmentation adaptively as the data resource is expanded.

Other kinds of large-Tb data sets are encountered in the natural language processing of genomic sequences arising from protein and DNA sequence languages in microbial genomes and microbiomes [63]. The goal is to use natural language models to help predict the functional categories of sequences, most of which are unannotated. Again, the same kind of design questions arise. What are the most informative functional categories to explore with annotation? How do we go about training, validating, and testing natural language models for genomic sequences? MINE would presumably help to select functional categories for discovery potential.

MINE is a discovery tool designed specifically for very large genetic data sets and is illustrated in a variety of problems within genetics here. MINE is built upon other ensemble methods [18] that have been developed for fitting models with *n* < < *p*. These ensemble methods of model identification coupled with MINE complete a discovery cycle (Fig. 6) for exploring problems in genetics. This discovery cycle has been called computing life [45]. MINE as a discovery tool completes the cycle in both analyzing and designing future costly omics experiments arising in genetics and allows an adaptive approach to solving problems in genetics.

**Key Points**

- New model-guided and adaptive experimental design for omics experiments called the Maximally Informative Next Experiment or MINE is reviewed for genetics.
- Ensemble methods are reviewed to fit models when the number of parameters *p* greatly exceeds the number of data points *n* (*p* > > *n*).
- MINE uses a fitted model ensemble for model-guided discovery when *p* > > *n* to select the next MINE, thereby better distinguishing models within a fitted model ensemble.
- MINE is illustrated in nonlinear models of the clock in *Neurospora crassa* in multiyear transcriptomics experiments.
- MINE is also illustrated in linear models for a genome-wide association study in *Sorghum bicolor* for multiyear experiments to find the genes underlying biomass.

## Supplementary Material

video1_bbaf167

## Data Availability

The data underlying this article will be shared on reasonable request to the corresponding author.
